# The association between glutathione reductase and postmenopausal osteoporosis: a retrospective study

**DOI:** 10.3389/fendo.2025.1679509

**Published:** 2026-01-02

**Authors:** Jili Wang, Kaixuan Wu, Menghan Chen, Xiaoyu Li, Saibo Ban, Bingbing Li, Kaiguang He, Huicai Yang, Qiuqing Dai, Shaochong Guo, Ziyuan Guo, Linyi Wang, Xuzhao Du, Dongliang Shi

**Affiliations:** 1 The Second Affiliated Hospital of Henan University of Chinese Medicine, Zhengzhou, China; 2 Henan Provincial Hospital of Traditional Chinese Medicine, Zhengzhou, China; 3 Henan University of Chinese Medicine, Zhengzhou, China

**Keywords:** postmenopausal osteoporosis, retrospective study, glutathione reductase, oxidative stress, adenosine deaminase

## Abstract

**Background:**

Oxidative stress is a common pathological condition in postmenopausal osteoporosis. Glutathione reductase, an antioxidant enzyme, plays a critical role in the body’s antioxidant defense system. However, the relationship between glutathione reductase and postmenopausal osteoporosis remains unclear. This study aims to explore the association between glutathione reductase and postmenopausal osteoporosis.

**Methods:**

This study retrospectively analyzed hospitalized postmenopausal women aged over 45 years from China between January 1, 2020, and April 15, 2025. The cases were divided into the osteoporosis group (OP group, T ≤ -2.5) and the non-osteoporosis group (non-OP group, T > -2.5). Initially, independent-samples t-tests were performed to compare differences in continuous variables between the two groups. Subsequently, binary logistic regression was conducted to identify potential predictors, followed by variable selection using the least absolute shrinkage and selection operator (LASSO) regression. The selected variables were then incorporated into a multivariable logistic regression model to determine independent risk factors. Finally, the predictive performance of the model was evaluated using receiver operating characteristic (ROC) curves, concordance index (C-index), calibration curves, and decision curve analysis (DCA).

**Results:**

A total of 401 patients were enrolled in this study, with 149 in the non-OP group and 252 in the OP group. The OP group showed lower BMI, lumbar spine (L1-L4), femoral BMD, and hip BMD compared to the non-OP group (P < 0.05). The OP group had higher age, adenosine deaminase (ADA), and glutathione reductase (GR) levels than the non-OP group (P < 0.05). ROC analysis revealed that the area under the curve for GR was 0.604 (95% CI: 0.556-0.662), with a critical value of 55.8 U/L (P < 0.05). Binary logistic regression and LASSO regression analyses demonstrated that GR ≥ 55.8 U/L was a risk factor for postmenopausal osteoporosis.

**Conclusion:**

GR are significantly associated with postmenopausal osteoporosis, and GR ≥ 55.8 U/L is an important risk factor for postmenopausal osteoporosis.

## 1 Introduction

Osteoporosis is a chronic, progressive, and clinically silent disease that significantly impacts public health, particularly among the elderly (1). In the United States, there are 10.2 million people with osteoporosis, and more than 2 million fractures occur each year due to osteoporosis. This condition severely compromises physical health and quality of life, especially in older populations. Treatment for osteoporosis often requires prolonged medication regimens, spanning 1–8 years, which imposes a substantial economic burden on patients and society (2). A cross-sectional study in China reported that a prevalence of osteoporosis of 5.0% in men and 20.6% in women aged 40 and older, highlighting postmenopausal osteoporosis as an escalating public health concern (3).

Oxidative stress, characterized by an imbalance between reactive oxygen species (ROS) production and antioxidant defenses, is a key pathological mechanism underlying numerous diseases, including heart failure, hypertension, diabetes, atherosclerosis, Parkinson’s disease, chronic kidney disease, and intervertebral disc degeneration (4–6). Oxidative stress disrupts bone homeostasis by suppressing osteoblast-mediated bone formation and promoting osteoclast-mediated bone resorption, leading to a net loss of bone mass (7, 8). Riegger et al. (9) have suggested that targeting mitochondrial dysfunction and oxidative stress could offer therapeutic potential for age-related degenerative diseases, including osteoarthritis and osteoporosis.

Glutathione reductase (GR) is a pivotal enzyme in the body’s antioxidant defense system, maintaining the reduced form of glutathione to neutralize reactive oxygen species (ROS). Kim et al. reported that enhanced GR expression confers antioxidant protection by preserving the cellular reduced glutathione/oxidized glutathione (GSH/GSSG) balance and mitigating apoptosis triggered by reactive oxygen species (10). Reduced serum GR levels have been associated with antioxidant defense failure and are proposed as a predictive marker for diseases such as COVID-19 (11). Conversely, studies have shown that inhibiting GR activity can modulate oxidative stress responses (12). León-Reyes G et al. suggested that reduced activity of antioxidant enzymes such as GR may contribute to increased mineral loss in bone tissue; however, GR activity remains largely stable in postmenopausal women with osteoporosis (13, 14). Faruk Sendur et al. (15) reported an association between decreased bone density and lower GR levels, while another study observed higher mean plasma GR activity in osteoporosis patients compared to healthy controls (16). These conflicting findings underscore the complexity of the relationship between GR and postmenopausal osteoporosis. This study aims to explore the potential association between GR levels and postmenopausal osteoporosis, providing insights into its role in disease pathogenesis and potential therapeutic implications.

## 2 Materials and methods

### 2.1 Ethical approval

This study has been approved by the Ethics Committee of Henan Provincial Hospital of Traditional Chinese Medicine (Second Affiliated Hospital of Henan University of Chinese Medicine), Ethical Approval No: HNSZYYYWZ-2025042.

### 2.2 Study subjects

This retrospective study analyzed postmenopausal women hospitalized between January 1, 2020, and April 15, 2025, at Henan Provincial Hospital of Traditional Chinese. The inclusion criteria for the study subjects were (1): postmenopausal women over 45 years old; (2) confirmed diagnosis of osteoporosis or non-osteoporosis via dual-energy X-ray absorptiometry (DXA); (3) availability of complete biochemical data on bone metabolism and liver function; (4) written informed consent obtained from the patients. Exclusion criteria included (1): presence of metabolic disorders such as thyroid dysfunction or hypopituitarism; (2) diagnosed hepatic dysfunction; (3) use of anti-osteoporosis medications within the preceding six months; (4) postmenopausal duration of less than one year. Patients were divided into two groups based on bone mineral density (BMD) measurements: the osteoporosis group (OP group, T-score ≤ -2.5) and the non-osteoporosis group (non-OP group, T-score > -2.5). The lowest T-score, obtained from either the lumbar spine or hip, was used for group classification.

### 2.3 Data collection

The following demographic characteristics were collected: age (years), height (cm), weight (kg), body mass index (BMI, kg/m²), as well as medical history including hypertension, diabetes, chronic kidney disease, cardiovascular diseases, respiratory diseases, stroke, and gastrointestinal diseases. Every subject included in the study received dual-energy X-ray absorptiometry (DXA) scan (Hologic Discovery Wi, model 010-0575, USA). Measured sites comprised lumbar spine bone mineral density (L1 BMD, L2 BMD, L3 BMD, L4 BMD, L1-L4 BMD, g/cm²), femoral neck BMD (Femoral neck BMD, g/cm²), and hip BMD (Hip BMD, g/cm²). Bone turnover markers were measured by electrochemiluminescence (Roche cobas e601, Switzerland), and additional biochemical indices were obtained with an automated analyzer (Abbott Architect c16000, USA). Laboratory biochemical indicators included: (1) bone metabolism markers (BMM) including osteocalcin (OCN, ng/mL), β-C-terminal telopeptide of type I collagen (β-CTx, pg/mL), total N-terminal propeptide of type I collagen (P1NP, pg/mL); (2) liver function tests including alanine aminotransferase (ALT, U/L), aspartate aminotransferase (AST, U/L), total protein (TP, g/L), albumin (ALB, g/L), globulin (GLO, g/L), total bilirubin (TBIL, μmol/L), direct bilirubin (DBIL, μmol/L), gamma-glutamyl transferase (GGT, U/L), alkaline phosphatase (ALP, U/L), total bile acid (TBA, μmol/L), cholinesterase (CHE, kU/L), prealbumin (PA, mg/L), adenosine deaminase (ADA, U/L), glutathione reductase (GR, U/L), and α-L-fucosidase (AFU,nmol/24h).

### 2.4 Statistical analysis

Continuous variables were expressed as means ± standard deviation. Differences between the OP group and the non-OP group were compared to using independent sample t-tests and chi-square tests. Pearson correlation analysis was performed to examine the correlation between ADA, GR, and BMD/BMM. ROC curve analysis was performed for ADA, GR, and other indicators, with results expressed by the area under the curve (AUC) and its 95% confidence interval (CI). Each point on the ROC curve represents the sensitivity and specificity of the indicator at a given threshold, from which an intermediate range between sensitivity and specificity was established to determine the optimal threshold for laboratory indicators. Binary logistic regression analysis was performed to design two models calculating the odds ratio (OR) and 95% CI for relevant experimental variables, to identify potential diagnostic biomarkers. Statistical analysis was conducted using R software (version 4.4.3). Some quantitative variables were converted into categorical variables based on cutoff values. To further verify the binary logistic regression results, the least absolute shrinkage and selection operator (LASSO) method was applied to select features with non-zero coefficients. Subsequently, multivariate logistic regression analysis was conducted to build a predictive model, incorporating variables selected by the LASSO regression model. Features in the multivariate logistic regression model were represented by regression coefficients (β), odds ratios (OR), 95% confidence intervals (CI), and P-values. All significance levels were two-tailed. A calibration curve was drawn to assess the calibration of the risk prediction nomogram. A significant Hosmer-Lemeshow test statistic (P < 0.05) indicated poor calibration, suggesting potential discrepancies between predicted probabilities and observed results, which could impact model validity. The C-index was calculated to quantify the discriminatory ability of the risk prediction nomogram. Bootstrap resampling (1000 times) was used to validate and calculate the adjusted C-index. Decision curve analysis (DCA) was used to evaluate the clinical utility of the risk prediction nomogram by quantifying the net benefit at different threshold probabilities in the female patient cohort. Net benefit was calculated by subtracting the proportion of false positives from the proportion of true positives, weighing the relative harm of missed intervention versus the negative consequences of unnecessary intervention. Statistical analysis, including independent sample t-tests and chi-square tests, was conducted using SPSS 26.0 (IBM Corp., Armonk, NY, USA), with P < 0.05 considered statistically significant.

## 3 Results

### 3.1 Baseline characteristics

In this study, there were 252 in the OP group and 149 in the non-OP group. Significant differences were observed between the two groups in age, height, weight, and BMI (P < 0.05). Specifically, the OP group was older and exhibited lower height, weight, and BMI compared to the non-OP group. No significant differences were found in the prevalence of underlying diseases between the two groups (P > 0.05) (**Table 1**).

**Table 1:** Demographic data between non-OP group and OP group.

Variables	Non-OP group	OP group	P value
Number	149	252	
Age (years)	58.6 ± 9.1	65.6 ± 8.9	P<0.001
Weight (kg)	64.7 ± 9.3	60.9 ± 8.7	P<0.001
Height (cm)	159.0 ± 6.2	156.8 ± 6.5	P<0.001
BMI (kg/m^2^)	25.5 ± 3.2	24.8 ± 3.4	0.031
hypertension	38	77	0.280
Diabetes mellitus	14	26	0.766
Chronic kidney disease	1	4	0.655
Cardiovascular disease	16	42	0.103
Respiratory disease	6	20	0.124
Stroke	6	16	0.324
Astrointestinal diseases	16	34	0.420

BMI, body mass index.

P < 0.05 was considered statistically significant.

### 3.2 Clinical laboratory biochemical indicators in postmenopausal osteoporosis and non-osteoporosis patients

**Figure 1:**
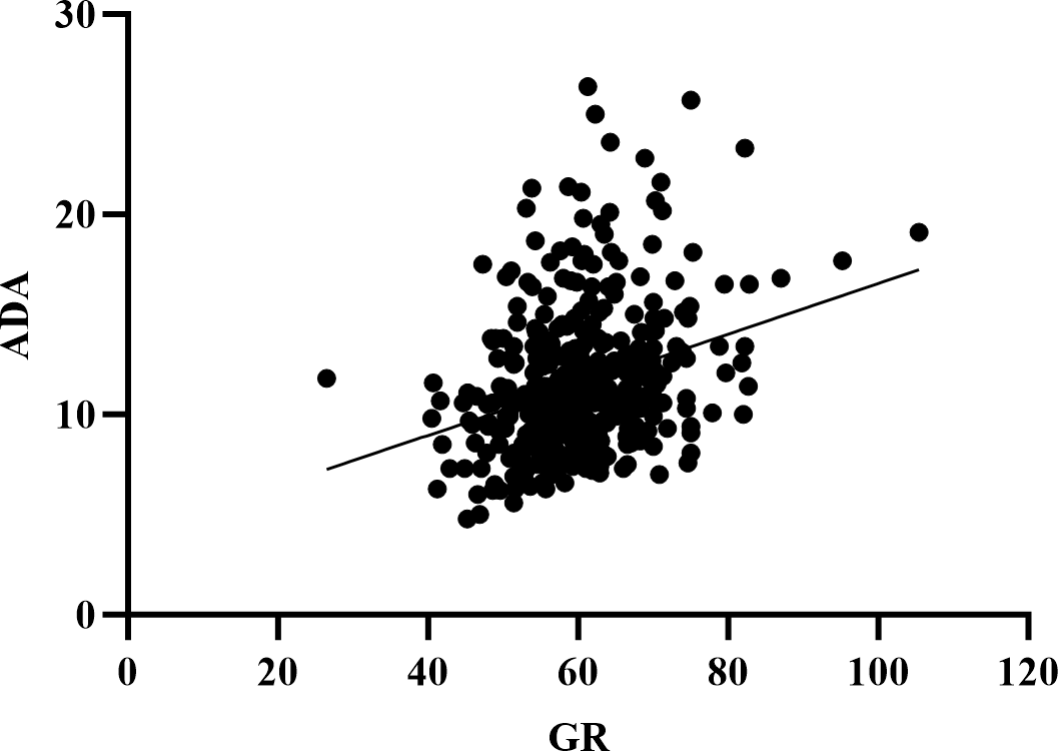
The relationship between GR and ADA. GR: glutathione reductase; ADA: adenosine deaminase.

The L1-L4 BMD, Femoral BMD, and Hip BMD in the OP group were lower than those in the non-OP group. Compared to the non-OP group, the both of ADA and GR were significantly higher in the OP (P < 0.05) (**Table 2**). Therefore, we conducted a correlation analysis of ADA and GR with BMD/BMM, as shown in **Table 3**: GR was negatively correlated with L2 BMD, L3 BMD, and L1-L4BMD (P < 0.05), and positively correlated with P1NP (P < 0.05); no statistically significant correlation was found between ADA and BMD/BMM. Moreover, ER showed a positive correlation with ADA (r=0.306, P< 0.05) (**Figure 1**).

**Table 2:** Clinical and laboratory characteristics between non-OP group and OP group.

Variables	Non-OP group	OP group	P value
L1 BMD (g/cm²)	0.9 ± 0.1	0.7 ± 0.2	P<0.001
L2 BMD (g/cm²)	0.9 ± 0.1	0.8 ± 0.2	P<0.001
L3 BMD (g/cm²)	1.0 ± 0.1	0.8 ± 0.1	P<0.001
L4 BMD (g/cm²)	1.0 ± 0.2	0.8 ± 0.1	P<0.001
L1–4 BMD (g/cm²)	0.9 ± 0.1	0.8 ± 0.1	P<0.001
Femoral neck BMD (g/cm²)	0.7 ± 0.1	0.6 ± 0.3	P<0.001
Hip BMD (g/cm²)	0.9 ± 0.1	0.7 ± 0.2	P<0.001
OCN (ng/mL)	18.7 ± 6.6	19.4 ± 8.4	0.165
β - CTx (pg/mL)	0.5 ± 0.3	0.5 ± 0.3	0.274
P1NP (pg/mL)	56.0 ± 26.7	59.6 ± 41.8	0.397
ALT (U/L)	19.4 ± 9.3	19.6 ± 14.7	0.843
AST (U/L)	20.6 ± 6.2	22.1 ± 9.4	0.061
TP (g/L)	68.6 ± 8.0	69.7 ± 5.0	0.100
ALB (g/L)	41.7 ± 4.5	41.1 ± 3.7	0.220
GLO (g/L)	28.0 ± 3.6	28.4 ± 3.8	0.341
TBIL (μmol/L)	12.1 ± 4.5	13.1 ± 5.1	0.062
DBIL (μmol/L)	4.4 ± 1.7	4.7 ± 1.9	0.157
GGT (U/L)	21.5 ± 15.1	23.1 ± 15.1	0.326
ALP (U/L)	76.3 ± 23.0	82.9 ± 40.0	0.070
TBA (μmol/L)	4.9 ± 4.1	5.3 ± 4.4	0.362
CHE (kU/L)	8.9 ± 1.5	8.9 ± 1.9	0.655
PA (mg/L)	238.0 ± 38.0	238.6 ± 45.7	0.883
ADA (U/L)	10.9 ± 3.4	11.8 ± 3.7	0.014
AFU (nmol/24h)	21.5 ± 4.4	21.5 ± 5.4	0.851
GR (U/L)	58.1 ± 7.7	61.4 ± 9.0	P<0.001

BMD, bone mineral density; OCN, osteocalcin; β-CTx β-collagen C-telopeptide; P1NP,total type I collagen amino-terminal propeptide; ALT, alanine aminotransferase; AST, aspartate aminotransferase; TP, total protein; ALB, albumin; GLO, globulin; TBIL, total bilirubin; DBIL,direct bilirubin; GGT, γ-glutamyl transferase; ALP, alkaline phosphatase; TBA, total bile acids; CHE, cholinesterase; PA, prealbumin; ADA, adenosine deaminase; GR, glutathione reductase; AFU, α-L-fucosidase.

P < 0.05 was considered statistically significant.

**Table 3:** Correlation analysis between BMD/BMM and laboratory variables.

Variables	L1 BMD	L2 BMD	L3 BMD	L4 BMD	L1–4 BMD	Femoral neck BMD	Hip BMD
GR	-0.073	-0.150*	-0.143*	-0.118*	-0.141*	-0.010	-0.074
ADA	-0.034	-0.061	-0.095	-0.029	-0.062	0.081	-0.094
	OCN			β - CTx		P1NP	
GR	-0.008			0.015		0.165*	
ADA	0.000			0.036		0.086	

ADA, adenosine deaminase; GR, glutathione reductase; BMD, bone mineral density.

*P < 0.05 was considered statistically significant.

Additionally, ROC analysis was performed to evaluate the diagnostic accuracy of the GR and ADA for osteoporosis. The optimal cutoff values for ADA and GR were as follows: ADA: 8.05 U/L (area under the curve: 0.567; P < 0.05; 95% CI: 0.508-0.625); GR: 55.8 U/L (area under the curve: 0.604; P < 0.05; 95% CI: 0.556-0.662). As shown in **Table 4**.

**Table 4:** Sensitivity analysis of laboratory with osteoporosis.

Variables	AUC	95% CI	P	Sensitivity	Specificity	Threshold
GR	0.604	0.556-0.662	0.029	0.758	0.436	55.8 U/L
ADA	0.567	0.508-0.625	0.025	0.893	0.208	8.05 U/L

ADA, adenosine deaminase; GR, glutathione reductase.

P < 0.05 was considered statistically significant.

### 3.3 Binary logistic regression analysis

Binary logistic regression was employed to examine the factors influencing osteoporosis, with the dependent variable being a binary outcome. In model 1, the independent variable was GR, while model 2 included continuous variables (such as GR, age, BMI, OCN, β-CTx, P1NP, ADA). Variables were selected through univariate analysis, and significant variables were incorporated into the multivariate model using the Enter method. The results are presented in **Table 5**: GR ≥ 55.8 U/L (model 1: OR: 2.226, 95% CI: 1.449-3.421, P < 0.001; model 2: OR: 2.450, 95% CI: 1.542-3.892, P < 0.001) was identified as an independent risk factor for postmenopausal osteoporosis.

**Table 5:** Binary logistic regression analysis results of factors of osteoporosis.

Factors	OR	95%	P
Model 1			
GR≥55.8 U/L	2.226	1.449-3.421	P<0.001
Model 2			
Age	1.077	1.050-1106	P<0.001
BMI	0.897	0.839-0.959	0.001
GR≥55.8 U/L	2.450	1.542-3.892	P<0.001

Model 1, GR;

Model 2, Age, BMI, OCN, β - CTx, P1NP, ADA, GR;

BMI, body mass index; OCN, osteocalcin; β-CTx, β-collagen C-telopeptide; P1NP, total type I collagen amino-terminal propeptide; ADA, adenosine deaminase; GR, glutathione reductase.

P < 0.05 was considered statistically significant.

### 3.4 LASSO regression analysis

**Figure 2:**
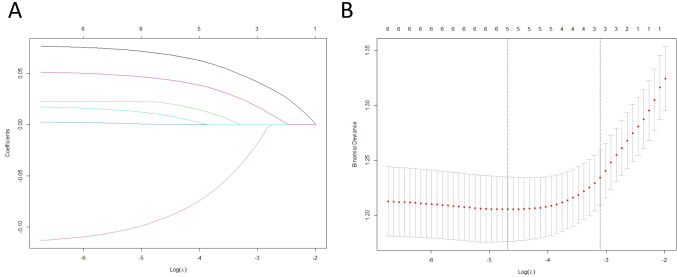
Selection and cross-validation analysis of key biomarkers for postmenopausal osteoporosis using LASSO. **(A)** LASSO coefficient spectrum for 6 features. **(B)** In the LASSO model, the optimal lambda is selected using ten-fold cross-validation with a minimization criterion.

**Figure 3:**
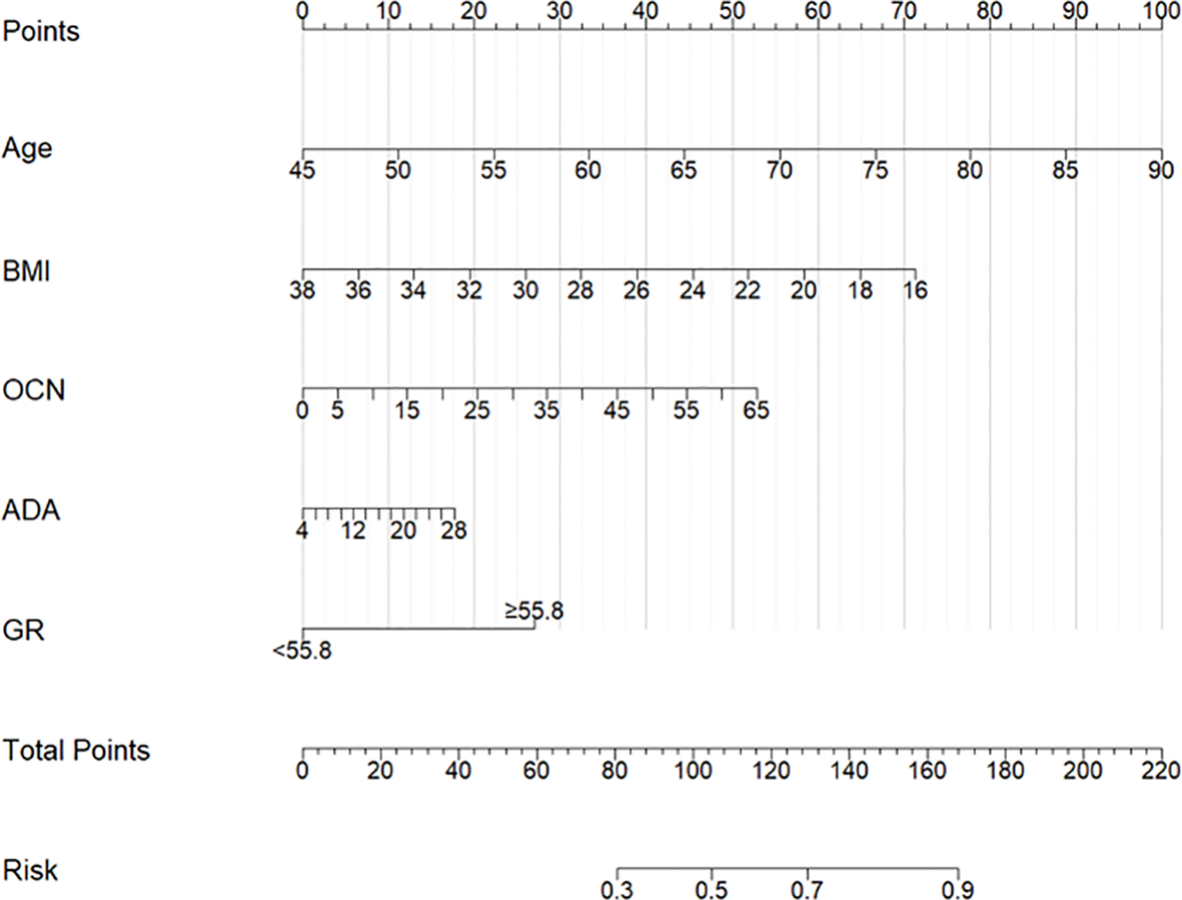
Nomogram for predicting postmenopausal osteoporosis risk. A Multi-Indicator Model Based on Age, BMI, OCN, ADA, and GR. BMI: body mass index; OCN: osteocalcin; ADA: adenosine deaminase; GR: glutathione reductase.

**Figure 4:**
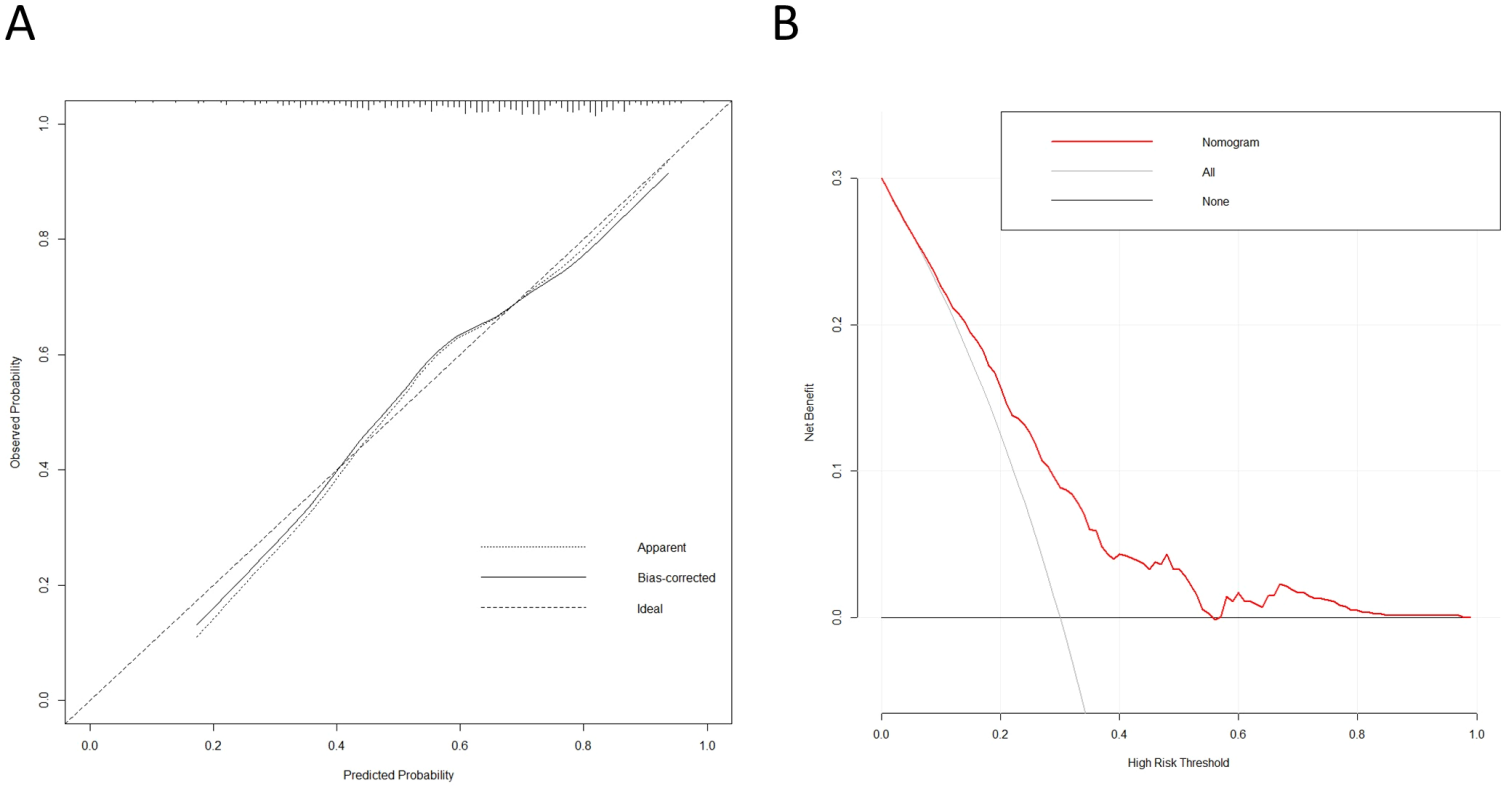
Calibration and clinical benefit of the osteoporosis risk nomogram. **(A)** Calibration curve for the predicted OP risk nomogram in the cohort. **(B)** Decision curve analysis for the OP risk nomogram.

To further confirm the robustness of the binary logistic regression model, we applied Lasso regression for penalized variable selection. Lasso regression, using 10-fold cross-validation, identified three features with non-zero coefficients (**Figures 1**, **2**), including age, BMI, and GR. These results were consistent with the findings from the binary logistic regression analysis, reaffirming that GR ≥ 55.8 U/L is an independent risk factor for postmenopausal osteoporosis, indicating that the model results are robust (**Figures 2**–**4**).

## 4 Discussion

BMD is typically reduced in osteoporosis patients. This study found that the clinical laboratory biochemical marker GR is significantly correlated with BMD, and GR ≥ 55.8 U/L is an important risk factor for postmenopausal osteoporosis.

GR, a commonly used indicator in osteoporosis detection, is closely associated with the disease’s pathogenesis. Previous studies have demonstrated that genetic variations in antioxidant response proteins, such as glutathione S-transferase, are associated with reduced BMD (17). Abdulabbas et al. (18) reported that antioxidant enzymes produced by bone cells, including glutathione reductase (GSR), neutralize free radicals and prevent the conversion of oxygen into hydroxyl radicals. Their findings suggest that GSR gene polymorphisms may reduce the risk of osteoporosis. Additionally, research has shown that nicotine and cotinine suppress the activities of catalase and GR, leading to increased ROS accumulation, which negatively impacts osteoblastic differentiation (19). These observations align with our findings, which indicate that GR mitigates oxidative stress and inhibits bone resorption. Furthermore, Liu et al. (20) found that superoxide dismutase (SOD) and GR are associated with the prognosis of hip fractures in elderly patients, supporting the role of antioxidants in reducing oxidative stress.

Contrary to previous reports of decreased GR in osteoporosis, we observed elevated serum GR in postmenopausal osteoporotic women. Several explanations may account for this divergence. Firstly, elevated GR levels may represent a compensatory response to increased oxidative stress, whereby the body up-regulates GR activity to maintain the intracellular GSH/GSSG ratio (10). However, in a chronic or excessive stress state such as PMOP, persistently elevated GR suggests that the antioxidant system is being pushed to its limits and that the oxidative burden exceeds the body’s compensatory capacity. In this context, higher GR levels are more appropriately regarded as a biomarker of oxidative imbalance and disease severity, rather than an effective protective factor. This interpretation is supported by the recent study of Cecerska-Heryć et al., who reported that COVID-19 is associated with a marked decrease in reduced GSH and disruption of redox homeostasis, accompanied by increased activities of GR and glutathione S-transferase (GST). This pattern reflects an adaptive, compensatory response to oxidative injury. Importantly, GR activity increased with age and correlated positively with mortality, indicating that GR functions as an age-sensitive biomarker of disease severity (21). Secondly, the stage of disease, subject characteristics and concomitant disorders may modulate whether GR activity rises or falls. For instance, Sontakke et al. (13) found comparable serum GR levels between postmenopausal osteoporotic patients and controls (patients: 41.31 ± 2.0 mmol/min/l; controls: 40.12 ± 3.2 mmol/min/l), with no significant difference. Yet their sample was small and markedly age-discrepant between groups. Subsequently, Sendur et al. (15) documented lower GR levels in postmenopausal osteoporotic patients than in healthy postmenopausal women (patients: 11.3 mol/min/gHb; controls: 13.2 mol/min/gHb), but their sample size was also smaller than ours, and they assayed erythrocyte GR whereas we measured serum GR, which may explain the divergent findings.

Our study identifies GR as a sensitive laboratory marker for postmenopausal osteoporosis. Specifically, women with GR levels ≥ 55.8 U/L exhibited a 2.226-fold increased likelihood of developing postmenopausal osteoporosis, underscoring a robust association between elevated GR levels and disease occurrence. To enhance the robustness of the analysis, LASSO regression was utilized to address multicollinearity and mitigate overfitting risks inherent in traditional stepwise regression by penalizing less significant predictors. This method identified age, BMI, and GR levels ≥ 55.8 U/L as key risk factors for osteoporosis. This approach effectively reduces the risk of overfitting commonly associated with traditional stepwise regression. Given its high sensitivity, GR represents a promising risk factor for osteoporosis, potentially aiding in the evaluation of drug efficacy by monitoring this biomarker. Further studies are required to elucidate the mechanisms underlying the interplay between GR, oxidative stress, and osteoporosis pathogenesis.

ADA is a key enzyme widely distributed in human tissues, capable of modulating immune responses, inflammation, and cellular metabolism. ADA is more commonly linked to rheumatoid arthritis (RA), with studies suggesting that RA patients are at an increased risk of secondary osteoporosis (21). Research by Shan Cao et al. (22) demonstrated that L-arginine mitigates inflammation-induced bone loss in arthritis, while ADA inhibitors abolished the suppressive effect of L-arginine on osteoclastogenesis in both *in vitro* and *in vivo* models. These findings suggest a complex role for ADA in bone metabolism that warrants further investigation. Increasing evidence indicates that elevated ADA activity is closely linked to immune-cell activation, pro-inflammatory cytokine production, and osteoclast differentiation. For example, Bhagavatham et al. showed that ADA triggers an inflammatory response in RAW264.7 macrophages while simultaneously promoting their differentiation into osteoclasts and suppressing the osteogenic differentiation of mesenchymal stem cells, thereby impairing their *in vivo* bone-forming capacity (23). In light of these findings, we now interpret the observed increase in ADA, together with its positive correlation with GR, as reflecting a coordinated interaction between immune activation and oxidative stress rather than a purely antioxidant response.

Additionally, this study found that ALP levels were higher in OP group compared to the non-OP group, but the difference was not statistically significant (P > 0.05). A study in the U.S. found that elevated serum total alkaline phosphatase (T-ALP) levels were significantly associated with lower BMD and increased osteoporosis risk in participants (24). It is important to note that ALP should not be used as a sole diagnostic criterion for osteoporosis. Ng E et al. (25) reported that persistently low serum ALP could indicate hypophosphatasia, which should be distinguished from osteoporosis to prevent misdiagnosis. These differences may require further exploration with larger sample sizes.

Moreover, our study found that serum OCN and β-CTx concentrations did not differ between the OP and control cohorts, while BMD readily separated them. Such discrepancy likely stems from the marked biological variability inherent to bone turnover markers. Filella et al. (26) summarized that OCN and β-CTx are readily influenced by diverse physiological and environmental determinants, generating pronounced inter- and intra-individual variability that can mask between-group contrasts. Seasonal illustration is provided by winter-related acceleration of bone turnover and consequent elevation of osteocalcin (27). Huo et al. (28) demonstrated that serum OCN and β-CTx decrease in spring, increase from summer onward, remain on an upward trajectory through autumn–winter, and reach their maximum in winter. In addition, turnover markers capture the dynamic facet of skeletal metabolism, while BMD offers an integrated read-out of chronic bone mass accrual and attrition. Consequently, the observed divergence between biochemical markers and BMD underscores that exclusive reliance on OCN/β-CTx inadequately portrays the skeletal condition of postmenopausal females.

This retrospective study is prone to selection and recall biases, which may affect the reliability of the findings. The limited sample size and reliance on data from a single institution constrain the generalizability of the results. In addition, turnover markers capture the dynamic facet of skeletal metabolism, while BMD offers an integrated read-out of chronic bone mass accrual and attrition on data from a single institution restrict the generalizability of the results. Furthermore, the lack of longitudinal follow-up and comprehensive patient evaluations, such as quality-of-life assessments, hinders the ability to establish causal relationships between biochemical markers and osteoporosis progression. Besides, we did not have data on serum 25(OH)D or PTH, which are major determinants of calcium-phosphate homeostasis and bone metabolism; therefore, we were unable to adjust for vitamin D status or secondary hyperparathyroidism, and residual confounding by these factors cannot be excluded. Future research should prioritize larger, multi-center, prospective studies to enhance the robustness and broader applicability of the findings, thereby informing more effective clinical guidelines.

## 5 Conclusion

This study identified a significant association between GR and postmenopausal osteoporosis. GR demonstrates high sensitivity and specificity for postmenopausal osteoporosis, with GR ≥ 55.8 U/L being a significant risk factor for the disease.

## Data Availability

The original contributions presented in the study are included in the article/supplementary material. Further inquiries can be directed to the corresponding authors.
